# Manipulation for treatment of degenerative lumbar spondylolisthesis: A protocol of systematic review and meta-analysis

**DOI:** 10.1097/MD.0000000000018135

**Published:** 2019-12-10

**Authors:** Kai Sun, Long Liang, He Yin, Jie Yu, Minshan Feng, Jiawen Zhan, Zhefeng Jin, Xunlu Yin, Xu Wei, Liguo Zhu

**Affiliations:** a Department of Spine, Wangjing Hospital of China Academy of Chinese Medical Sciences; b Beijing Key Laboratory of Orthopedics of Traditional Chinese Medicine; c Department of Rehabilitation; d Office of Academic Research, Wangjing Hospital of China Academy of Chinese Medical Sciences, Beijing, China.

**Keywords:** degenerative lumbar spondylolisthesis, effectiveness and safety, manipulation, randomized controlled trials, systematic review

## Abstract

**Background:**

Degenerative lumbar spondylolisthesis (DLS) is one of the common orthopedic diseases which causes low back pain in patients, which seriously affects people's daily life and work. As a method of conservative treatment of this disease, manipulation is widely used in clinical practice. We will summarize the current published evidence of manipulation in the treatment of DLS, and evaluate the effectiveness and safety of manipulation through systematic review and meta-analysis, so as to provide more reliable evidence for future clinical practice.

**Methods:**

We will conduct a comprehensive search of the following 9 databases until January 2019: PubMed, Embase, Cochrane Library, ClinicalTrials.gov, Web of Science, Chinese National Knowledge Infrastructure, Chinese Science and Technique Journals Database, Wan Fang Database, and Chinese Biomedical Database. The 2 researchers will independently search, screen, extract data, and evaluate the quality of the literatures. The primary outcomes include clinical effectiveness, Japanese Orthopaedic Association scores, and the secondary outcomes include visual analog scale scores, symptom scores, and adverse events. Bias risk tools provided by Cochrane Collaboration will be used for literature quality assessment, and RevMan 5.3 software will be used for meta-analysis.

**Results:**

The results of this study will systematically evaluate the effectiveness and safety of manipulation intervention for people with DLS, especially in improving lumbar function scores and pain scores.

**Conclusion:**

The systematic review of this study will summarize the current published evidence of manipulation for the treatment of DLS, which can further guide the promotion and application of it.

**Ethics and dissemination:**

This study does not require ethical approval and the results will be published in a peer-reviewed journal.

**PROSPERO registration number:**

CRD42019139933.

## 1 Introduction

Degenerative lumbar spondylolisthesis (DLS) is one of the common orthopedic diseases that causes low back pain, radiation pain in the affected limbs, and intermittent claudication.^[1,2]^ It is generally believed that the initial event comes from disc degeneration, and most cases are at L4-L5. Then, because of long-term sustained lumbar instability or stress increase, the corresponding small joints begin to wear, resulting in DLS.^[3–5]^ The disease is more common in middle-aged and older people over the age of 50, in which the incidence of women is higher than that of men. The prevalence ratio of gender is about 1.3:1.^[6]^ With the aging of the world population and the accelerated pace of people's lives, the number of patients with DLS is increasing year by year. Because of its risk of disability, it seriously reduces the quality of patients’ life and has become one of the chronic refractory diseases that plagues people's daily life and work.

Both surgical and conservative methods can be used for the treatment of this disease.^[2,7]^ Surgery can effectively alleviate clinical symptoms of patients by using simple decompression, minimally invasive surgery, fusion techniques, etc, especially for patients who have slipped more than III°.^[8–10]^ However, surgery has a lot of problems such as wound infection, postoperative recurrence, and high cost. Therefore, we need to strictly control the indications for surgery.^[11,12]^ Conservative treatments include methods such as drug therapy, manipulation, and traction. For most patients, conservative treatments should be considered 1st, regardless of whether they have pain symptoms due to nerve compression.^[2,13,14]^ Spinal manipulation therapy is an important part of conservative therapy. Current published evidence suggested that it is widely used in clinical practice.^[15–17]^ Moreover, many scholars have carried out a series of studies on the mechanism of manipulation in the treatment of DLS from the perspective of spinal biomechanics, the relationship between spinal canal and dural sac, and the 3-dimensional motion of lumbar spine.^[18–20]^

Although the present research on the effectiveness and safety of manual treatment of DLS is increasing, there is no systematic review and meta-analysis specifically for this. So based on this issue, we will evaluate the effectiveness and safety of manipulation through systematic review and meta-analysis, to provide more reliable evidence for future clinical practice.

## 2 Methods

### 2.1 Design and registration

This study has been registered in the Prospective Register of Systematic Reviews (PROSPERO) (no: CRD42019139933; http://www.crd.york.ac.uk/PROSPERO). The meta-analysis will be developed in compliance with the Preferred Reporting Items for Systematic Reviews and Meta-Analyses (PRISMA) approach and reported adhering to the guidelines.^[21]^

### 2.2 Criteria for considering studies for this review

#### 2.2.1 Types of studies

All relevant prospective randomized controlled trials (RCTs) will be conducted to evaluate the effectiveness and safety of manipulation for DLS as alternative treatment. Case reports, non-RCTs, quasi-RCTs, animal or cell experiments, and other studies will be excluded.

#### 2.2.2 Types of participants

Patients with a clear diagnose of DLS will be included, and the race, sex, duration of disease, and age of the patients are not limited.

#### 2.2.3 Types of interventions

The intervention in the experimental groups was manipulation or manipulation plus other measures. All manual-related procedures should be included, such as manipulation, manual therapy, chiropractic, tuina, and massage. However, studies that compare the effectiveness or safety of different forms of manipulation will be excluded as this is different from the focus of this study. The control groups which can verify the effectiveness or safety of the manipulation as a monotherapy or in combination with conventional therapies will be considered. And the control group should be nondrug therapy that does not include the manipulation.

#### 2.2.4 Types of outcome measures

Clinical effectiveness, Japanese Orthopaedic Association scores are the primary outcomes, and visual analog scale scores, symptom scores, and adverse events are the secondary outcomes. The clinical effectiveness standard is according to the Clinical Trials of New Patent Chinese Medicines.^[22]^

### 2.3 Search methods for the identification of studies

#### 2.3.1 Data sources

A comprehensive search strategy will be carried out within the following databases including PubMed, Embase, the Cochrane Library, ClinicalTrials.gov, Web of Science, Chinese National Knowledge Infrastructure, Chinese Science and Technique Journals Database, Wan Fang Database, and Chinese Biomedical Database. We will also retrieve any other gray literature sources. There will be no limitation on language, publication type and status.

#### 2.3.2 Search strategy

The following terms will be used in the search: “Spondylolisthesis,” “Degenerative spondylolisthesis,” “Lumbar spondylolisthesis,” “Manipulation,” “Chiropractic,” “Manual therapy,” “Chiropractic,”“Tuina,” and “Massage.” The actual search strategies will be as follows: PubMed database: (((((spondylolisthesis[MeSH Terms]) OR spondylolisthesis[Title/Abstract]) OR Spondylolistheses[Title/Abstract]) OR Spondylistheses[Title/Abstract])) AND ((((((Chiropractic[Title/Abstract]) OR manipulation[Title/Abstract]) OR massage[Title/Abstract]) OR tuina[Title/Abstract]) OR manual therapy[Title/Abstract]) OR manipulation[MeSH Terms]).

### 2.4 Study selection

**Figure 1:**
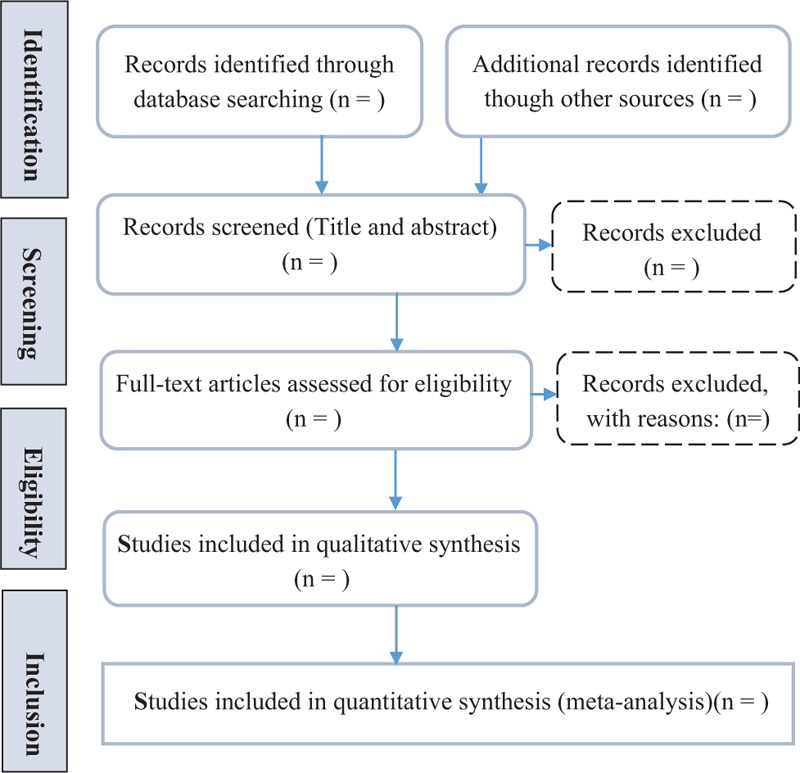
Flow diagram of study selection process.

Two researchers (SK and LL) will independently search the database of the literature, and will be independently screen according to inclusion and exclusion criteria. Subsequently, the 2 researchers will cross-check the selection results. We will use NoteExpress V.3.2.0 software for document management. Any differences between the researchers will be resolved through discussion with a third researcher's opinion (WX and ZL). The details of the selection process will be presented in a PRISMA flow diagram (Fig. 1).

### 2.5 Data extraction and management

According to the characteristics of the study, we will prepare an excel form for data collection before data extraction. Two researchers (SK and LL) will independently work for data extraction. The main extracted information is as follows: the publication time of the literature, the name of the 1st author, gender, age, interventions in the experimental and control groups, courses of treatment, outcomes, follow-up, side effect, and so on. The above information will be finally cross-checked by 2 researchers (SK and LL) and a 3rd trained researcher (YH or WX) will help to check and solve any disagreements occurred between 2 researchers.

### 2.6 Quality assessment

We will use the bias risk tool provided by the Cochrane Collaboration^[23]^ to evaluate the quality of the literature using RevMan software (V.5.3). The main evaluation includes 7 entries: random sequence generation, allocation concealment, blinding of participants and personnel, blinding of outcome assessment, incomplete outcome data, selective reporting, and other sources of bias. According to the above 7 items, the 3 levels of low risk, high risk, and uncertain risk will respectively assess the risk of bias in the literature. As with the previous process, it will be independently assessed by 2 researchers. If there is disagreement, it will be discussed with the 3rd researcher (ZL or WX).

### 2.7 Data analysis

RevMan software (V.5.3) will be utilized for data analysis in our study. For continuous results, we will use the mean difference with 95% confidence intervals (CIs) for representation. For the dichotomous results, we will calculate the relative risk and 95% CIs for each outcome. *I*^2^ statistic and Chi-squared test will be used to assess potential heterogeneity in the studies. The fixed effect model will be used if *I*^2^ ≤ 50%, *P* > .1. When *I*^2^ > 50% and *P* < .1, we will analyze data using a random-effects model.

### 2.8 Sensitivity analysis and subgroup analysis

In addition, if there is significant heterogeneity, we will use sensitivity analysis or subgroup analysis to find the cause of the heterogeneity. Subgroup analysis usually explores the source of heterogeneity from the perspective of clinical heterogeneity and methodologic heterogeneity. We can divide the subgroup into groups according to population characteristics, intervention methods, treatment time, and so on. Sensitivity analysis will mainly delete each of the included studies to determine whether they will have a specific impact on the results of meta-analysis.

### 2.9 Reporting bias analysis

If there are more than 10 qualified studies are included in our study, funnel plots and Egger regression analysis will be carried out to assess the publication bias.

### 2.10 Ethics and dissemination

This study does not require ethical approval and the results will be published in a peer-reviewed journal.

## 3 Discussion

As a kind of commonly and frequently global disease in the middle aged and elderly, DLS causes a great deal of burden socially and financially.^[1–2,6]^ As we know, manipulation therapy has attracted more and more attention from patients with DLS. Kovanur-Sampath et al's^[24]^ study showed that manipulation can play a therapeutic role by changing biochemical markers. Several previous studies have reported that manipulation can treat DLS effectively and safely.^[15–17]^ It may relieve the patient's clinical symptoms by relaxing the muscles, regulating the disorder of the facet joints, and adjusting the stress distribution.^[25–27]^ Unfortunately, its effectiveness and safety for treating this disorder is still inconclusive.

Therefore, we will summarize the current published evidence to assess the effectiveness and safety of manipulation for the treatment of patients with DLS. But inevitably, this systematic review may have some shortcomings, such as race, age, gender, intervention, and the diversity of manipulation treatment processes, which may lead to higher clinical and statistical heterogeneity. In short, we hope to provide more information for doctors and patients through these studies.

## Author contributions

**Conceptualization:** Kai Sun, Long Liang, He Yin.

**Data curation:** Zhefeng Jin, Xunlu Yin.

**Investigation:** Jiawen Zhan, Minshan Feng.

**Methodology:** Xu Wei, Liguo Zhu.

**Resources:** Zhefeng Jin, Xunlu Yin.

**Software:** Jie Yu, Jiawen Zhan.

**Supervision:** Jie Yu, Minshan Feng.

**Writing – original draft:** Kai Sun, Long Liang, He Yin.

**Writing – review & editing:** Xu Wei, Liguo Zhu.
